# Voxel-wise body composition analysis using image registration of a three-slice CT imaging protocol: methodology and proof-of-concept studies

**DOI:** 10.1186/s12938-024-01235-x

**Published:** 2024-04-13

**Authors:** Nouman Ahmad, Hugo Dahlberg, Hanna Jönsson, Sambit Tarai, Rama Krishna Guggilla, Robin Strand, Elin Lundström, Göran Bergström, Håkan Ahlström, Joel Kullberg

**Affiliations:** 1 Radiology, Department of Surgical Sciences, Uppsala University, Uppsala, Sweden; 2 Department of Information Technology, Uppsala University, Uppsala, Sweden; 3 Department of Molecular and Clinical Medicine, Institute of Medicine, Sahlgrenska Academy, University of Gothenburg, Gothenburg, Sweden; 4 Department of Clinical Physiology, Sahlgrenska University Hospital, Region Västra Götaland, Gothenburg, Sweden; 5 Antaros Medical, Mölndal, Sweden

**Keywords:** Image registration, Computed tomography, Body composition, Imiomics analysis

## Abstract

**Background:**

Computed tomography (CT) is an imaging modality commonly used for studies of internal body structures and very useful for detailed studies of body composition. The aim of this study was to develop and evaluate a fully automatic image registration framework for inter-subject CT slice registration. The aim was also to use the results, in a set of proof-of-concept studies, for voxel-wise statistical body composition analysis (Imiomics) of correlations between imaging and non-imaging data.

**Methods:**

The current study utilized three single-slice CT images of the liver, abdomen, and thigh from two large cohort studies, SCAPIS and IGT. The image registration method developed and evaluated used both CT images together with image-derived tissue and organ segmentation masks. To evaluate the performance of the registration method, a set of baseline 3-single-slice CT images (from 2780 subjects including 8285 slices) from the SCAPIS and IGT cohorts were registered. Vector magnitude and intensity magnitude error indicating inverse consistency were used for evaluation. Image registration results were further used for voxel-wise analysis of associations between the CT images (as represented by tissue volume from Hounsfield unit and Jacobian determinant) and various explicit measurements of various tissues, fat depots, and organs collected in both cohort studies.

**Results:**

Our findings demonstrated that the key organs and anatomical structures were registered appropriately. The evaluation parameters of inverse consistency, such as vector magnitude and intensity magnitude error, were on average less than 3 mm and 50 Hounsfield units. The registration followed by Imiomics analysis enabled the examination of associations between various explicit measurements (liver, spleen, abdominal muscle, visceral adipose tissue (VAT), subcutaneous adipose tissue (SAT), thigh SAT, intermuscular adipose tissue (IMAT), and thigh muscle) and the voxel-wise image information.

**Conclusion:**

The developed and evaluated framework allows accurate image registrations of the collected three single-slice CT images and enables detailed voxel-wise studies of associations between body composition and associated diseases and risk factors.

**Supplementary Information:**

The online version contains supplementary material available at 10.1186/s12938-024-01235-x.

## Introduction

Metabolic diseases, such as type 2 diabetes (T2D) and cardiovascular diseases (CVD), are some of the leading causes of mortality globally; existing protocols of prevention and diagnosis may be obsolete [1].

Body composition is known to be an important risk factor associated with both T2D and CVD. Different methods are used to study body composition, including imaging techniques such as computed tomography (CT). CT images provide detailed analysis, which can aid in understanding the relationship between body composition and these diseases [2, 3].

The Swedish Cardiopulmonary Bioimage Study (SCAPIS) and Impaired Glucose Tolerance Microbiota (IGT) studies were established to thoroughly investigate the existing protocols of prevention and diagnosis related to metabolic diseases such as T2D and CVD, with the aim of improving them. These nationwide studies incorporate advanced imaging technologies, biomarkers, and epidemiological analyses to study more than 30,000 individuals. SCAPIS and IGT include CT imaging based on 3-single-slice (liver, abdomen, and thigh) protocols.

The SCAPIS study [1] intends to use imaging techniques to investigate fat deposits in conjunction with clinical data as well as data obtained through “omics” technologies to improve our understanding of the role of obesity and diabetes, associated with CVD and chronic obstructive pulmonary disease (COPD).

Similarly, the objective of the IGT study [4] is to investigate the impact of the gut microbiota on glucose dysregulation and the development of CVD.

CT scans are commonly used in medical image analysis as they can provide high-resolution anatomical information of the whole body. Diagnosis, treatment planning and evaluation of disease progression can be done through CT images [5–7]. To better understand large-scale image data, it is important to transform them into a common geometry before analyzing them. Image registration plays a significant role in image analysis, especially in the realm of medical imaging. It can be used to improve the accuracy and reliability of image analysis methods by deforming images into a common reference space [8].

Image registration techniques are typically tailored for the imaged body region and imaging modality they intended to be used for. As described in [9, 10], the authors used to identify a spatial transformation that aligns a collection of images into a common reference space that helps to fuse images acquired by different modalities. They investigated the anatomical and structural changes in longitudinal studies and extended their study to conduct a statistical voxel-wise body composition analysis [11–13].

To utilize the full potential of the CT images in the SCAPIS and IGT studies, there is a need for efficient image registration methods prior to applying an Imiomics analysis (association between imaging and non-imaging data) [13] to the 3-slice CT images.

The use of deep learning (DL) based registration methods has increased in recent years due to their faster runtime and ability to avoid issues specific to the optimization process of classical methods [14]. However, these methods often require large datasets to produce reliable results, and newer methods without this requirement have only been tested on limited datasets [15, 16]. There are still several domain specific challenges, which need to be addressed before DL-based image registration methods can be widely used in clinical settings instead of classical methods [17].

Previously, image registration methods have used combinations of rigid and deformable steps [13, 18–20] or biomechanical models [21, 22]. However, only a limited amount of research on 3-slice CT image registration has been conducted**,** and also most of the research has focused on registering serial CT images from a maximum of 30 subjects [19]. Inter-subject 3D CT image registration has previously been studied in the context of atlas generation from 1466 lung screening CT images. This approach used masks of lung and body to guide and evaluate the registrations [23]. As per our knowledge, inter-subject image registration of 3-slice CT into common space, for subsequent voxel-wise statistics, is unexplored.

The aim of this study was to develop and evaluate a fully automated inter-subject registration technique for a 3-slice CT imaging protocol, allowing detailed voxel-wise studies of associations between imaging and non-imaging data. An image registration method that leverages the CT images in parallel with segmentation masks was developed and evaluated using more than 8000 images from two cohorts. Results were evaluated using common registration evaluation metrics and proof-of-concept studies where prior information on what voxel-wise results to expect were available.

## Results

The results of the image registration followed by Imiomics analysis are presented in this section, and the visual representation of registered images is illustrated in Additional file 1: Fig S1. Table 1 presents an overview of the performance metrics for the quality of the transformations for the 3-slice CT images.

**Table 1:** Comparison of performance metrics for 3-slice CT registration methods

Cohort	Slice	Sex	Subjects	VME (mm)	IME (HU)	Number of folds	Registration time (s)^a^
SCAPIS	Liver	M	500	2.41 ± 1.28	37.28 ± 7.3	0.16 ± 0.5	5
		F	453	2.47 ± 1.61	33.24 ± 5.28	0.18 ± 0.45	4.5
	Abdomen	M	502	2.84 ± 1.53	48.67 ± 5.85	1.03 ± 5.29	6.5
		F	455	2.75 ± 1.79	45.41 ± 5.49	0.97 ± 7.4	7
	Thigh	M	502	1.97 ± 0.86	29.08 ± 4.36	0.64 ± 4.52	5
		F	455	1.33 ± 0.73	28.81 ± 4.23	0.49 ± 2.04	5.5
IGT	Liver	M	784	2.04 ± 0.92	31.11 ± 7.04	0.14 ± 1.46	4.5
		F	990	2.15 ± 1.03	32.82 ± 5.52	0.59 ± 1.12	4
	Abdomen	M	812	2.99 ± 1.23	49.7 ± 6.87	0.91 ± 6.09	7.5
		F	1011	2.73 ± 1.06	43.24 ± 6.94	0.71 ± 4.43	6.5
	Thigh	M	812	1.29 ± 0.87	30.91 ± 5.89	0.82 ± 6.51	5.5
		F	1009	1.11 ± 0.56	26.04 ± 4.91	0.53 ± 8.89	6

Data are presented as mean ± STD for vector magnitude error (VME), intensity magnitude error (IME), and Hounsfield unit (HU)

^a^Only mean was measured

The evaluation revealed that thigh registration yielded the best performance, while liver registration transformations were the quickest to complete. The abdomen registration process, on the other hand, comparatively, took a longer time and gave higher vector magnitude error (VME) and intensity magnitude error (IME) scores.

**Fig. 1:**
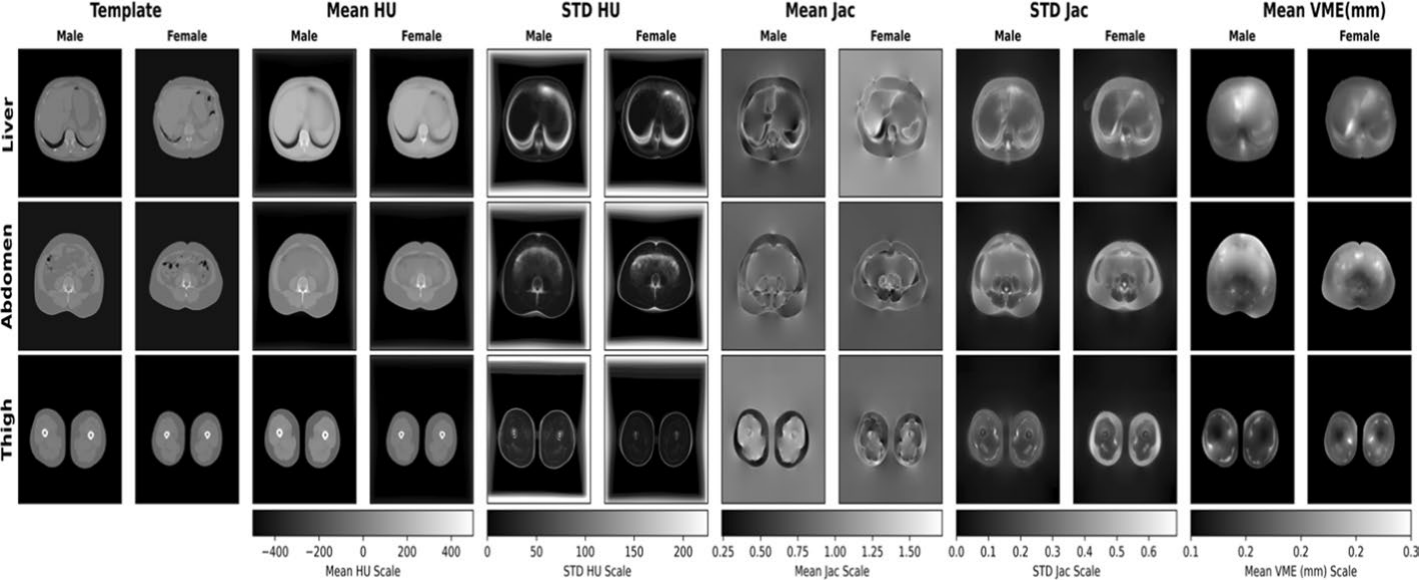
Resulting CT scans collage of males (*n* = 502) and females (*n* = 455) from the SCAPIS cohort study. The template images are the preprocessed images, while the mean, standard deviation (STD) intensity and mean vector magnitude error (VME) of all registered images are shown as Mean HU, STD HU and VME, respectively. The mean and STD of the logarithm Jacobian in every point are illustrated in the mean Jac and STD Jac, respectively

Voxel-wise statistics results of image registration are illustrated in Fig. 1. The Jacobian mean image of the liver slice, displayed in the figure, clearly highlights the presence of the liver, spleen, lungs, vertebra, and SAT. However, the visualizations of the ribs are obscure, and the anterior left side appears darker and blurrier. The standard deviation (STD) of high intensity Hounsfield unit (HU) is primarily concentrated in the lungs, air and vertebra. In contrast, the liver, spleen, and subcutaneous fat exhibited a low-intensity STD.

The visualization of the average VME indicates that the registration exhibits the least inverse consistency at the upper right edge of both the male and female liver and at the bottom edge of the female liver, mainly due to the presence of the kidneys in the female temple. High VME values are observed at the boundary between the spleen and lungs, while lower VME values are found in the middle of the liver, the upper part of the vertebra, and the SAT.

Further analysis of the average Jacobian, calculated for all points in Fig. 1, where a lower value signifies local contraction and a higher value indicates local expansion, demonstrates an overall higher average. This suggests that the template images are generally smaller than the other images in the data. Notably, the mean Jacobian for males is lower at the bottom of the liver and higher at the bottom of the spleen, indicating that the template image has larger lungs on one side than the target (moving) images. The female collage has a higher Jacobian mean in the lungs, indicating that the template images generally have a lower lung than the target images. Also, the female mean Jacobian has lower values at the bottom of the liver, indicating the presence of kidneys. The lungs have a high Jacobian STD, indicating that the size of the lungs varies between subjects in the dataset. The male mean Jacobian of liver and spleen has a lower value as compared to the female mean Jacobian, which also shows that the male template has larger liver and spleen volumes. Notably, adipose tissue consistently exhibits a higher Jacobian STD rate than liver, spleen and bone marrow, indicating greater variability in its size among subjects.

The mean of the registered male and female abdomen images depicted in the figure appears sharp in the skeletal muscles, vertebrae, and SAT but blurry in the intra-abdominal region. This region also exhibits a high STD and a white border at the top and bottom. The most anterior skeletal muscles display the least consistent registration, while the most posterior SAT exhibit the most consistent registration. The mean Jacobian reveals that the lateral SAT, skeletal muscles are generally larger in the template images (lower value). The SAT, intra-abdominal region, anterior skeletal muscle and intestinal gases have high Jacobian standard deviations, indicating that their size varies greatly between subjects. In contrast, skeletal muscles and vertebrae display lower deviations, suggesting that their sizes remain relatively consistent across subjects.

For the thigh slice, the mean images of male and female collages are sharp, with clear distinctions between lean tissue, adipose tissue, and bone. Notably, the only high STDs are found at the boundaries between tissue types. The average VME is low around the bone but higher in lateral SAT and at the boundary between adipose tissue and lean tissue. The mean Jacobian of the male thigh is lower in the lateral SAT, indicating more adipose tissue in the template images, and higher in the lean tissue, indicating less lean tissue in the template images. For the female mean, Jacobian is lower in lean tissue than adipose tissue, which shows that there is more lean tissue than adipose tissue in female temples. The Jacobian STD in the female thigh is higher in the lateral SAT than the lean tissue, indicating more variation between images in the lateral SAT. However, in the male thigh, Jacobian STD is lower in lateral SAT and lean tissue, indicating less variation between images, and higher at the border of lean tissues, indicating higher variation.

**Fig. 2:**
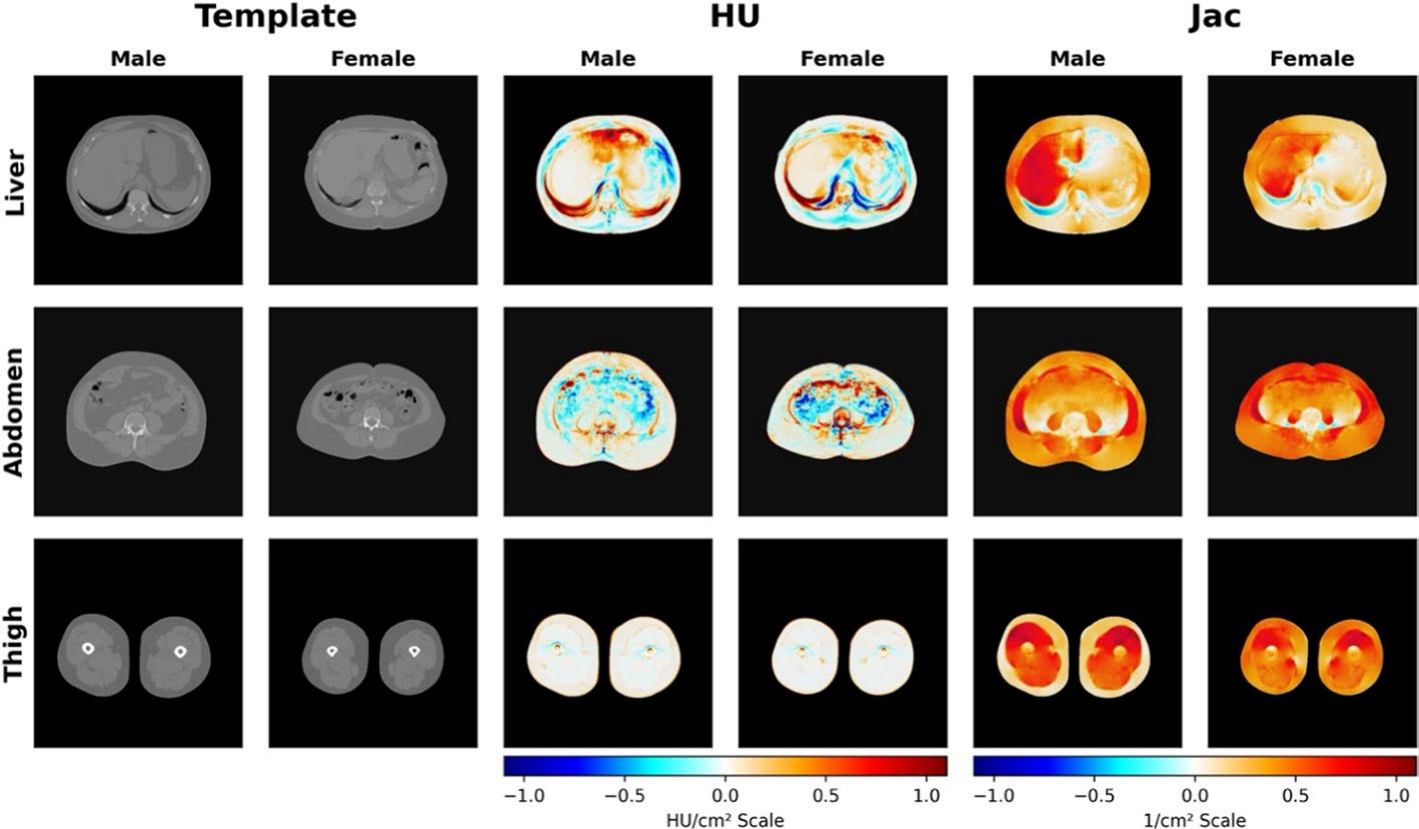
Resulting Imiomics collage for 3-slice CT images of (liver, abdomen, and thigh). From left to right, selected processed CT template images, deformed Hounsfield unit’s images, and Jacobian determinant images are represented for all male (*n* = 502) and female (*n* = 455) subjects, from the SCAPIS cohort. The collage Imiomics (deformed HU and Jac determinant) images were associated with non-imaging data. The 3-slice (liver, abdomen, and thigh) collage shows voxel-wise regression results (beta values) between liver area for liver slice, skeletal muscle area for abdominal slice, and thigh muscle area for thigh slice

Deformed CT images were further employed to conduct a voxel-wise analysis of associations between the CT images (as represented by tissue volume from HU and Jacobian determinant) and various explicit measurements collected in both cohort studies, as depicted in Fig. 2. The utilization of Imiomics analysis enabled the examination of associations between various segmentation measurements (liver, spleen, abdominal muscle, VAT, SAT, thigh SAT, IMAT, and thigh muscle) and other associated information. As illustrated in Fig. 2 and Additional file 1: Fig S3, the results indicate a positive correlation between 3-slice CT images of the liver, abdomen, and thigh organs and non-imaging parameters, such as liver area for the liver slices, skeletal muscle area for the abdominal slices, and thigh muscle area for the thigh slices, which were measured explicitly with the assistance of previously trained UNET++ [24] based deep learning models. The voxel-wise statistical analysis was carried out by employing linear regression models to determine the relationship between imaging and non-imaging data, as demonstrated by the HU/cm^2^ and Jac/cm^2^ scales. The findings from the analysis indicated that the images exhibited a close relationship with the expected outcomes (with a slope close to 1). These results were visually displayed through color-coded representations, where regions were highlighted in different colors.

Additional file 1: Figs. S2, S4 show the resultant Imiomics collages for 3-slice CT voxel-wise associations with liver fat HU, spleen, VAT, SAT, IMAT, and thigh SAT.

Similarly, Additional file 1: Fig S5 presents the correlation matrices, which demonstrate the association of each variable with others and provide additional evidence of the Imiomics analysis by accurately highlighting the region of the correlation map.

## Discussion

In this study, we developed an image registration framework that successfully registered CT images from a 3-slice CT body composition analysis protocol from two large cohorts. The images were registered for males and females separately. The registration results were evaluated and further utilized to conduct voxel-wise regression analysis between imaging and non-imaging data proof-of-concept studies, where prior knowledge of what results to expect was available.

Our developed image registration framework allows inter-subject-based detailed and precise voxel-wise analysis of body composition in large study populations, as well as a cohort saliency analysis from deep regression studies [25, 26]. The registration method utilizes a one-step approach with a tissue-specific regularization weight map. The technique utilizes multi-channel input, including CT images and generated masks of structures, in order to register, i.e., identify point-to-point-correspondence between subjects and avoid local minima. The performance of the registration was found to be good, see Table 1, with an average VME being similar to, or lower than, that from similar methods [12, 13].

In this study, the binary masks were found useful for image registration. However, we found that the best results were achieved when we assigned higher weights to the CT images compared to the individual masks. This is likely because the masks contribute with some high-level features, whereas the CT image contains more detailed information (spanning also the full images/or both spatially and intensity wise), which is useful to register the images into a common space.

For the liver slices, the visualization of the mean and STD of the registered male and female subjects demonstrated successful registration for most of the reference space (illustrated in Fig. 1). This is indicated by the relative sharpness of tissues and organs in the mean HU image. However, some areas of the image may not be perfectly registered. This is likely due to large inter-subject structural variability. Overall, the average VME is low, indicating consistent and high-quality registrations. The Jacobian mean image of female shows that the template images are generally smaller than the other images in the dataset, suggesting that the temple is not a precise mean image. This could potentially be improved by using different template images or synthesizing new template images using for example the method proposed by Pilia et al. [27]. The Jacobian STD also shows that the different tissues vary to different extents, with lungs, adipose tissue and bone/vertebra varying the most and liver and spleen varying the least.

For the abdominal slices, the visualization of the mean and STD of the registered male and female images shows a relatively sharp mean image and a low-intensity STD HU image in most areas (illustrated in Fig. 1). This indicates successful registration in most tissues, including skeletal muscles, vertebrae and abdominal SAT. However, the intra-abdominal region in the mean HU image is blurry, indicating less successful registration in this area. The high HU intensity STD in the anterior intra-abdominal region is due to differences in the amount and location of intestinal gases, and the large difference in HU between gas and tissue in CT. The relatively high average VME in most anterior skeletal muscles may be due to the vague or absent anterior skeletal muscles in some subjects, which affects the automatic generation of ISAT masks and the performance of the registration framework. However, the overall VME is low, indicating good quality in the produced transforms. The Jacobian values are mostly around one, with a mean value close to one, indicating that the template image is relatively close to representing an average shape of the dataset. The Jacobian STD of both males and females shows that the size of air between internal organs and adipose tissue varies the most between subjects, while lean tissue varies to some degree.

Similarly, for the thigh slices, the sharp mean HU images and the low-intensity STD (illustrated in Fig. 1) for both males and females indicate successful registration. The relatively high average VME at the lateral SAT, the border between lean tissue and adipose tissue may indicate uncommon tissue shapes in those regions. However, the registration is generally consistent, especially around the bone, which is in roughly the same place in different subjects and therefore does not require large deformations. The average of the Jacobian of the male thigh registrations shows that the template images have more SAT and less lean tissue in the dataset. Female thigh registration shows that template images have on average less SAT and lean tissue. The Jacobian STD of the thigh registration shows that the size of SAT varies more in females than in males, and lean tissue varies less in both.

From the voxel-wise Imiomics analysis of SCAPIS (Fig. 2), liver area is seen to correlate positively to liver size and attenuation for both male and female. The correlation was confirmed in Additional file 1: Fig S5. In the abdominalen slice the muscle area measurements are seen to correlate negatively to the intra-abdominal Hounsfield units (HUs). This is likely because abdominal muscle area correlates positively to VAT area (see Additional file 1: Fig S5). A larger VAT area will after the registration be compressed into the intra-abdominal region of the template image. Partial volume and the very challenging registration problem may result in that, on average, HUs are lower for persons with larger muscle area.

In a previous study including 3D chest CT scans (*n* = 1466) inter-subject image registration methodology has been presented and evaluated [23]. The authors presented a standardized thoracic atlas to support lung cancer screening efforts, addressing the challenge of anatomical variability. They developed a multi-stage registration pipeline optimized for the entire thoracic space and included handling scans with missing information due to variations in FOV. The study aimed to create a resource for standardizing chest CT analysis for lung cancer screening and identifying phenotypic variations. In comparison with our work, both studies utilize image registration techniques; however, our work analyzes a specific three-slice CT imaging protocol and explores voxel-wise analysis for detailed body composition studies. The method presented in this work also contains a multi-channel approach that simultaneously uses the original CT images and tissue segmentation masks to improve the registration.

There are certain limitations to our study. One such limitation is that the requirement of segmentation masks for image registration may limit the applicability of the proposed method in certain contexts. In addition, another limitation is finding the best regularization settings (weights), and the fact that template images need to be selected. In this study, we selected template images separately for both sex and study since we assumed this would simplify the registration problem. To optimize the registration parameters, we utilized various metrics, including mean, standard deviation, and inverse consistency images, to evaluate the registrations. Proof-of-concept studies were also used to guide the optimization. It is important to acknowledge that the use of multiple reference spaces for the various body sections may result in fragmentation of the voxel-wise statistical analysis, potentially hindering the ability to draw comprehensive conclusions across studies and sexes. Another limitation of this study is, that the body composition's association with diseases and more risk factors remain untested. This we aim to address in future work.

## Conclusion

In this study, we have presented and evaluated an inter-subject image registration framework and proof-of-concept voxel-wise correlations using two cohort studies, SCAPIS and IGT. The proposed technique utilizes both low-level and high-level image features to achieve accurate registration, resulting in average VME and IMEs of less than 3 mm and 50 HUs, respectively. The proposed approach allows effective visualization of associations at the voxel level, with potential applications in studies of body composition and its relations to disease or disease risk factors. In the future, image registration can also be used to combine saliency information from deep regression analysis.

## Materials and methods

### Subjects and CT imaging

This study incorporates CT imaging data from two large-scale cohorts: SCAPIS and IGT. SCAPIS [1] is a study focusing on CVD and COPD in which 30,154 men and women participants aged between 50 and 64 years were voluntarily participating. The imaging data were collected from six university hospitals in Sweden over the years 2013 and 2018, and a random sample of the population recruited in Gothenburg was used in this study. Similarly, IGT [4] is a mirror cohort to SCAPIS, with its primary focus on individuals at risk of developing T2D and investigating the impact of the gut microbiota on glucose dysregulation and the development of CVD. The IGT study included 1,965 subjects with varying forms of glucose dysregulation and employed the same CT protocols as SCAPIS. Both studies were approved by the Swedish Ethical Review Authority (Dnr 2021-05856-01), and all participants provided written informed consent.

CT images were acquired using a Somatom Definition Flash with a Stellar detector (Siemens Healthcare, Forchheim, Germany) according to a specified procedure, with a 500 mm field of view (FOV), image dimensions of 512 × 512 × 1, and 5 mm slice thickness. The image slice locations of the liver, abdomen and thigh slices were determined as follows: the liver slice was reconstructed from the lung scan. The lung scan was performed during maximum inhalation, where the images could be rescanned once if needed. Reconstructed slices were selected with the goal of containing as much as possible of the liver, including right and left liver lobes, the spleen (a 2 cm^2^ region of interest (ROI) should be possible to place in the spleen), and some lung tissue. Instructions were also given to try to avoid the kidneys and the heart in the selected slice. If not possible to follow all instructions, the liver and spleen instructions were prioritized. The scanning was performed with kV and mAs settings of 120 kV, 25 mAs for the lung scan (liver slice), 120 kV and 40 mAs for the abdominal slice and 120 kV and 20 mAs for the thigh. Reconstruction was performed using kernel I31f medium smooth for the liver slice and B31s medium smooth for the abdominal and thigh slices. The abdominal single-slice scan was placed above the crista edge and in the center of vertebra L4. Preferably, no liver or kidneys should be seen in the slice. The priority was to avoid crests in the image. Regarding the thigh slice, it was taken in position midway between the outer edge of the acetabulum and the joint surface of the knee joint.

A total of 8285 slices (52.8% females) from the baseline visits from both studies with analyzable image quality and content as well as complete data on non-imaging and explicit measurements from the CT images were successfully included in this study. These included 2867 SCAPIS slices (47.5% females) and 5418 IGT slices (55.6% females). An overview of the datasets and their characteristics is presented in Table 2.

**Table 2:** Characteristics of SCAPIS and IGT cohorts

Characteristics/Cohort	SCAPIS		IGT	
Sex	Male	Female	Male	Female
Subjects/Slices	502/1504	455/1363	812/2408	1011/3010
Age (years)	58.33 ± 4.33	58.54 ± 4.23	58.34 ± 4.49	57.86 ± 4.54
BMI (kg/m^2^)	27.92 ± 4.16	27.16 ± 5.37	28.18 ± 3.95	27.37 ± 4.65

Age and BMI (mean ± STD)

The explicit measurements were quantified with the assistance of previously trained DL-based segmentation UNET ++ models [24]. The models exhibited Dice scores during cross-validations (liver = 0.994, spleen = 0.993, skeletal muscle = 0.988, visceral and subcutaneous adipose tissue (VAT and SAT) = (0.973 and 0.990), thigh muscle = 0.996, thigh SAT = 0.992, intermuscular adipose tissue (IMAT = 0.927).

### Image preprocessing

The initial preprocessing steps for image registrations involved setting a common physical origin for all images.

According to the CT acquisition protocol, the images should have a FOV of 500 mm [1]. However, there were a few images with other FOVs. These were resampled to the common physical dimensions. Images containing at least 50 voxels with intensities above 2000 HU were flagged for review to check for the presence of metallic implants. Intensity values above 1024 HU were truncated to 1024 HU, to remove outliers and irrelevant variation in extreme values between subjects.

### Binary masks

In parallel with CT images, binary masks were used for different preprocessing steps and to support the image registration process. The binary masks were created either by classical methods or using deep learning-based segmentations (UNET++ model [24, 28]). The classical methods used thresholding and basic image analysis approaches as described below.

A body mask for all slices of liver, abdomen and thigh was created by thresholding HU intensities above −190, labeling, selecting, and closing the largest object, using binary fill operations (for the thighs, the two largest objects). The body masks were employed to eliminate the CT table and other non-body objects to generate the processed CT images.

The vertebra masks, for the liver and abdominal slices, were created by use of thresholding (above 200 HU [29]) followed by morphological opening operations.

Similarly, for thigh registration, the cortical bone and lean tissue masks were generated by thresholding the intensity values (above 200 HU [29] for bone and between −29 HU and 150 HU [30] for lean tissue masks), labeling the largest object, and then extracting it from the mask in order to exclude the bone marrow. The bone marrow region inside the cortical bone was filled in order to reduce the number of local minima during registration.

Binary masks for liver, spleen, and abdominal muscles were created using deep learning as previously described as DL pipeline [24]. A few liver slices where the spleen was not visible (out of protocol) were excluded.

The abdominal muscle mask was used in combination with the abdominal slice in order to produce an inside subcutaneous adipose tissue (ISAT) mask with a similar technique as used [12].

### Registration methods

**Fig. 3:**
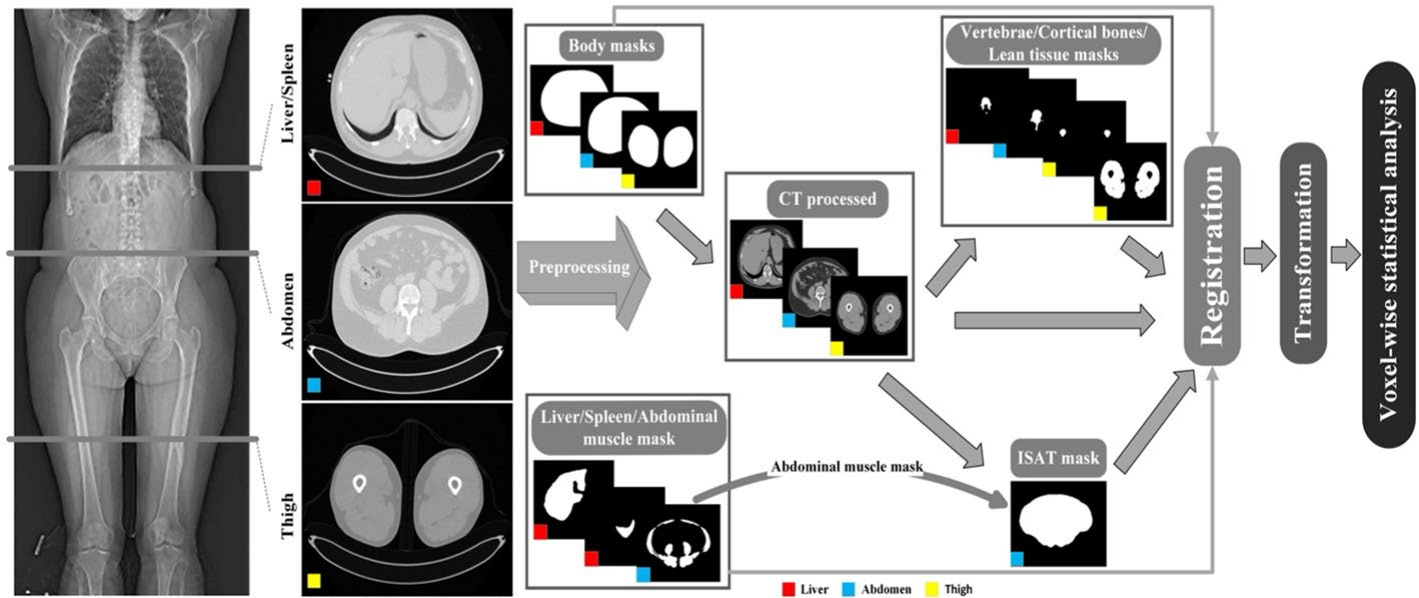
Proposed methodology for 3-slice CT (computed tomography) image registration to enable voxel-wise analysis. Registration was done slice-wise, small boxes in colored red, light blue and yellow represent each slice registration process which is performed independently. The process involved the generation of body masks for each slice from raw CT images. Processed CT images are the CT images after removal of CT tables and non-body objects, generated from raw CT images and body masks. Deep learning segmentation models were used to generate liver, spleen, and abdominal muscle segmentation masks. For creating ISAT (inside subcutaneous adipose tissue) masks the processed CT images and a predicted deep learning abdominal muscle mask were used. Lean tissue, vertebrae, and cortical bone masks were generated from processed CT images. All preprocessed images and masks were used simultaneously to perform registration tasks

The image registration procedure is described in Fig. 3.

The registration process of 3-slice CT images was done in a single step using a multi-channel input that includes the preprocessed CT images and the corresponding masks for each slice, as shown in Fig. 3. Our registration method relied on both a combination of low-level and high-level features to establish point-correspondence between complex and significantly different images. The CT image was set to a higher weight than the corresponding masks, which makes the registration rely more on the CT image than on the masks. Registration parameters and image weights are listed in Table 3, chosen based on preliminary testing on the sample.

**Table 3:** Image registration parameters

Parameter	Settings
Step size/block size	0.5/[16]
Update rule	Additive
Pyramid levels	8
Pyramid stop level	1
﻿Regularization weight map	[0.05,0.05,0.05,0.1] (air, adipose tissue, soft tissue, bone)

Weights	Liver	Abdomen	Thigh
CT image	0.5	0.4	0.5
**Masks**			
Liver	0.1	–	–
Spleen	0.1	–	–
Body	0.15	0.1	0.1
Vertebra	0.15	0.2	–
Skeletal muscle	–	0.2	–
ISAT	–	0.1	–
Bone	–	–	0.2
Lean tissue	–	–	0.2

The registration method was optimized using an objective function known as the sum of squared differences (SSD) and the least restricted transformation model to enable local displacements and create a displacement field.

A tissue-specific regularization weight map during registration was applied to each slice based on HU threshold, with lower weights for tissues with high elasticity (e.g., fat) and higher weights for tissues with low elasticity (e.g., bone).

A fast graph-cut based technique [31] and a hierarchical multi-resolution strategy with Gaussian image pyramids were used to optimize the objective function and avoid local minima.

The multi-resolution strategy commenced at the lowest resolution and ended at level one; the final resolution of the registered images was the original resolution downscaled by a factor of two in each dimension.

### Template image selection

The image registration is performed for the two studies and male and female subjects separately. Prior to utilizing the methods, a reference space or template image was selected. We used a methodology based on image segmentation results (z-scores) to select images corresponding to a representative “mean image”. The template image was selected by minimizing the sum of absolute *z*-scores for a set of parameters. Images were then visually quality controlled to ensure they had no major artifact or other unusual features.

For the liver slice, spleen, liver, abdominal visceral and subcutaneous adipose tissue areas were used. For the abdominal slice, abdominal VAT, SAT, and skeletal muscle areas were used. Similarly, for thigh slices, thigh SAT and thigh muscle were used to compute the sum of absolute z-score. We selected the template image with the lowest summed z-score value for both studies and sexes individually.

### Voxel-wise body composition analysis

The image registrations were utilized to conduct a set of voxel-wise body composition proof-of-concept studies. Voxel-wise linear regressions were performed between image information (intensities in terms of HUs and size differences by use of Jacobian determinants) and a set of explicit proof-of-concept measurements (e.g., explicit measurements area (in terms of cm^2^) and intensity measurements (HU) from the CT images).

Performing this linear regression for each pixel or voxel in a slice of the 2D image can provide insights into the relationship between the areas of interest and the corresponding voxel intensities in the CT image, allowing to understand how changes in the explicit measurement (e.g., liver area/abdominal VAT area) affect the voxel intensities.

To calculate the correlation map between image voxel intensities and non-imaging parameters (explicit measurements). The statistical linear regression method was used to model the relationship between image voxel intensity ${y}_{i,j,k}$ and explicit measurement ${x}_{i,j}$ as follows:

$${y}_{i,j,k}={\beta }_{0,j}+ {\beta }_{1,j }{x}_{i,j} + {\epsilon }_{i,j,k},$$

where $${\beta }_{0,j}=\mathrm{ intercept}, {\beta }_{1,j}= {\text{slope}}, {\epsilon }_{i,j,k} \sim N\left(0,{\sigma }^{2}\right),$$ and $${\sigma }^{2} =\mathrm{ error \ variance}.$$  

In this equation, ${y}_{i,j,k}$ is the predicted (correlation map) voxel intensity for voxel (i,j) with intensity ${x}_{i,j}$ in channel k (grayscale 1D), ${\beta }_{0,j}$ is the intercept (or bias term) for the linear regression model specific to the column or area j, ${\beta }_{1,j}$ is the slope (or coefficient) corresponding to ${x}_{i,j}$ and ${\epsilon }_{i,j,k}$ represents the error term or residual.

The error term ${\epsilon }_{i,j,k}$ is assumed to follow a normal distribution $N(0,{\sigma }^{2})$ with mean 0 and variance ${\sigma }^{2}$.

### Method evaluation

The image registration method was evaluated using three parameters: the transformed images, the quality of the transforms, and the computation time. The transformed images were evaluated for similarity with the template image through visual inspection and by calculating statistical measures such as the voxel-wise mean, and standard deviation (STD). The quality of the transforms was analyzed using inverse consistency (IC) and their diffeomorphic properties. To analyze the IC, reverse direction registrations were conducted, followed by an assessment of the average vector magnitude error (VME) and intensity magnitude error (IME) across the segmented body region. Subsequently, the Jacobians of the resultant transformations were computed.

The IC errors were defined in terms of average VME and IME for each segmented body region Ω in a pair of images (*x* and *y*). The intensity value of image x at each voxel location *i* was represented by ${I}_{x}(i)$. The IC errors for composite transforms ${T}_{xy}$ ◦ ${T}_{yx}$ and ${T}_{yx}$ ◦ ${T}_{xy}$ were averaged into a single value for each pair of registered images:

$${{\text{IME}}}_{xy}=\frac{1}{\left|\Omega \right|}\sum_{i\in \Omega }\parallel {I}_{x}\left(i\right)-{I}_{x}\left({T}_{yx} \circ {T}_{xy} \left(i\right)\right)\parallel$$

$${{\text{VME}}}_{xy}=\frac{1}{\left|\Omega \right|}\sum_{i\in \Omega }\parallel x-{T}_{yx} \circ {T}_{xy} (i))\parallel$$

The diffeomorphic property was evaluated by counting the number of points where the Jacobian of the resulting transforms was less than zero (number of folds). The natural logarithm of the Jacobian was computed to efficiently interpret local tissue volume differences between subjects. The computation time was measured for all preprocessing steps, excluding the time for generating explicit masks with the help of a deep learning models.

To assess the efficacy of the voxel-wise body composition analysis method, we performed both a visual analysis of the output results as well as an analysis of voxel-wise proof-of-concept regression studies.

In addition to evaluating the methods, a linear correlation analysis using the Pearson correlation coefficient was conducted to evaluate the association between non-imaging data (explicit measurement), which demonstrates the relationship of each variable with others.

The experiments were carried out on a Linux-based operating system with the following specifications: Intel (R) Xeon (R) W-2133 CPU at 3.60 GHz, 32 gigabytes (GB) of RAM, and Nvidia GeForce RTX 2080 Ti Graphic Card with 11 GB RAM.

### Supplementary Information

**Additional file 1: Figure S1** Visual representations of liver, abdomen, and thigh slices of templates (reference images), targeted images (moving or deforming images), and registered images (transformed images).** Figure S2.** Resulting Imiomics collage for 3-slice CT images of (liver, abdomen, and thigh). From left to right, selected processed CT template images, deformed Hounsfield unit, and Jacobian determinant images are represented for all male (n=502) and female (n = 455) subjects, from the SCAPIS cohort. The collage Imiomics (deformed HU and Jac determinant) images were associated with non-imaging data. The Hounsfield unit (HU) and Jacobian (Jac) collage show voxel-wise regression results (beta values) between a 3-slice image (liver, abdomen, and thigh) and the corresponding liver fat measurements in HU units. The subsequent HU and Jac images display correlations between liver CT slice and spleen area in cm^2^, abdomen CT slice and VAT area in cm^2^, thigh CT slice and thigh IMAT area in cm^2^. Similarly, in the last set of HU and Jac images, correlations between liver CT slice and abdominal SAT area in cm², abdomen CT slice and abdominal SAT area in cm^2^, thigh CT slice and thigh SAT area in cm^2^ are represented. Small colored boxes of green, red and white in each image indicating non imaging correlation measurements. abd is short for the abdomen.** Figure S3** Resulting Imiomics collage for 3-slice CT images of (liver, abdomen, and thigh). From left to right, selected processed CT template images, deformed Hounsfield unit’s images, and Jacobian determinant images are represented for all male (n=812) and female (n=1011) subjects, from the IGT cohort. The collage Imiomics (deformed HU and Jac determinant) images were associated with non-imaging data. The 3-slice (liver, abdomen, and thigh) collage shows voxel-wise regression results (beta values) between liver area for liver slice, skeletal muscle area for abdominal slice, and thigh muscle area for thigh slice.** Figure S4.** Resulting Imiomics collage for 3-slice CT images of (liver, abdomen, and thigh). From left to right, selected processed CT template images, deformed Hounsfield unit, and Jacobian determinant images are represented for all male (n = 812) and female (n = 1011) subjects, from the IGT cohort. The collage Imiomics (deformed HU and Jac determinant) images were associated with non-imaging data. The Hounsfield unit (HU) and Jacobian (Jac) collage show voxel-wise regression results (beta values) between a 3-slice image (liver, abdomen, and thigh) and the corresponding liver fat measurements in HU units. The subsequent HU and Jac images display correlations between liver CT slice and spleen area in cm², abdomen CT slice and VAT area in cm², thigh CT slice and thigh IMAT area in cm^2^. Similarly, in the last set of HU and Jac images, correlations between liver CT slice and abdominal SAT area in cm², abdomen CT slice and abdominal SAT area in cm^2^, thigh CT slice and thigh SAT area in cm^2^ are represented. Small colored boxes of green, red and white in each image indicating non imaging correlation measurements. abd is short for the abdomen.** Figure S5** Correlation (Pearson correlation coefficient) matrix of non-imaging variables (explicit measurements in cm^2^ and HU attenuation) for male and female participants (n = 8285) in SCAPIS and IGT studies. Measurements are in terms of area (cm^2^), and liver fat in terms of Hounsfield unit. abd is short for the abdomen.

## Data Availability

The SCAPIS dataset will be accessible to researchers, with the requirement that the principal investigator is currently affiliated with an institution in Sweden. Access will be granted through the data sharing platform following the completion of ethical approval and the submission and approval of a project application. IGT data may be provided for research collaborations upon reasonable request to the co-principal investigator, G.B.

## References

[1] Bergström G, Berglund G, Blomberg A, Brandberg J, Engström G, Engvall J, Eriksson M, Faire U, Flinck A, Hansson MG, Hedblad B, Hjelmgren O, Janson C, Jernberg T, Johnsson Å, Johansson L, Lind L, Löfdahl C-G, Melander O, Östgren CJ, Persson A, Persson M, Sandström A, Schmidt C, Söderberg S, Sundström J, Toren K, Waldenström A, Wedel H, Vikgren J, Fagerberg B, Rosengren A (2015). The Swedish CArdioPulmonary BioImage Study: objectives and design. J Intern Med.

[2] Seabolt LA, Welch EB, Silver HJ (2015). Imaging methods for analyzing body composition in human obesity and cardiometabolic disease: imaging methods for analyzing body composition. Ann NY Acad Sci.

[3] Xu K, Khan MS, Li TZ, Gao R, Terry JG, Huo Y, Lasko TA, Carr JJ, Maldonado F, Landman BA, Sandler KL (2023). AI body composition in lung cancer screening: added value beyond lung cancer detection. Radiology.

[4] Molnar D, Björnson E, Larsson M, Adiels M, Gummesson A, Bäckhed F, Hjelmgren O, Bergström G (2023). Pre-diabetes is associated with attenuation rather than volume of epicardial adipose tissue on computed tomography. Sci Rep.

[5] Al-Sharify ZT, Al-Sharify TA, Al-Sharify NT, Yahya naser H (2020). A critical review on medical imaging techniques (CT and PET scans) in the medical field. IOP Conf Ser Mater Sci Eng..

[6] Hricak H, Brenner DJ, Adelstein SJ, Frush DP, Hall EJ, Howell RW, McCollough CH, Mettler FA, Pearce MS, Suleiman OH, Thrall JH, Wagner LK (2011). Managing radiation use in medical imaging: a multifaceted challenge. Radiology.

[7] Raman SP, Mahesh M, Blasko RV, Fishman EK (2013). CT scan parameters and radiation dose: practical advice for radiologists. J Am Coll Radiol.

[8] Modersitzki J, Heldmann S, Papenberg N (2015). Nonlinear registration via displacement fields. Brain mapping.

[9] Maintz JBA, Viergever MA (1998). A survey of medical image registration. Med Image Anal.

[10] Hajnal JV, Hill DLG (2001). Medical image registration.

[11] Sotiras A, Davatzikos C, Paragios N (2013). Deformable medical image registration: a survey. IEEE Trans Med Imaging.

[12] Jönsson H, Ekström S, Strand R, Pedersen MA, Molin D, Ahlström H, Kullberg J (2022). An image registration method for voxel-wise analysis of whole-body oncological PET-CT. Sci Rep.

[13] Strand R, Malmberg F, Johansson L, Lind L, Sundbom M, Ahlström H, Kullberg J (2017). A concept for holistic whole body MRI data analysis. Imiomics PLoS ONE.

[14] Boveiri HR, Khayami R, Javidan R, Mehdizadeh A (2020). Medical image registration using deep neural networks: a comprehensive review. Comput Electr Eng.

[15] Haskins G, Kruger U, Yan P (2020). Deep learning in medical image registration: a survey. Mach Vis Appl.

[16] Fu Y, Lei Y, Wang T, Curran WJ, Liu T, Yang X (2020). Deep learning in medical image registration: a review. Phys Med Biol.

[17] Chen X, Diaz-Pinto A, Ravikumar N, Frangi A (2020). Deep learning in medical image registration. Prog Biomed Eng.

[18] Li X, Yankeelov TE, Peterson TE, Gore JC, Dawant BM (2008). Automatic nonrigid registration of whole body CT mice images: automatic registration of whole body CT images. Med Phys.

[19] Akbarzadeh A, Gutierrez D, Baskin A, Ay MR, Ahmadian A, Alam NR, Lövblad K, Zaidi H (2013). Evaluation of whole-body MR to CT deformable image registration. J Appl Clin Med Phys.

[20] Baiker M, Staring M, Löwik CWGM, Reiber JHC, Lelieveldt BPF, Fichtinger G, Martel A, Peters T (2011). Automated registration of whole-body follow-up MicroCT data of mice. Medical image computing and computer-assisted intervention—MICCAI 2011.

[21] Li M, Miller K, Joldes GR, Doyle B, Garlapati RR, Kikinis R, Wittek A (2015). Patient-specific biomechanical model as whole-body CT image registration tool. Med Image Anal.

[22] Li M, Miller K, Joldes GR, Kikinis R, Wittek A (2016). Biomechanical model for computing deformations for whole-body image registration: a meshless approach. Int J Numer Method Biomed Eng..

[23] Xu K, Gao R, Khan M, Bao S, Tang Y, Deppen S, Huo Y, Sandler K, Massion P, Heinrich MP, Landman BA. Development and characterization of a chest CT atlas. In: Landman BA, Išgum I. editors. Medical Imaging 2021: Image Processing. p. 48. SPIE, Online Only, United States, 2021. 10.1117/12.2580800 PMC8442827 34531633

[24] Ahmad N, Strand R, Sparresäter B, Tarai S, Lundström E, Bergström G, Ahlström H, Kullberg J (2023). Automatic segmentation of large-scale CT image datasets for detailed body composition analysis. BMC Bioinformatics.

[25] Langner T, Martínez Mora A, Strand R, Ahlström H, Kullberg J (2022). MIMIR: deep regression for automated analysis of UK Biobank MRI Scans. Radiol Artif Intell..

[26] Langner T (2021). Uncertainty-aware body composition analysis with deep regression ensembles on UK Biobank MRI. Comput Med Imag Graph..

[27] Pilia M, Kullberg J, Ahlström H, Malmberg F, Ekström S, Strand R (2019). Average volume reference space for large scale registration of whole-body magnetic resonance images. PLoS ONE.

[28] Zhou Z, Rahman Siddiquee MM, Tajbakhsh N, Liang J, Stoyanov D, Taylor Z, Carneiro G, Syeda-Mahmood T, Martel A, Maier-Hein L, Tavares JMRS, Bradley A, Papa JP, Belagiannis V, Nascimento JC, Lu Z, Conjeti S, Moradi M, Greenspan H, Madabhushi A (2018). UNet++: a nested U-Net architecture for medical image segmentation. Deep learning in medical image analysis and multimodal learning for clinical decision support.

[29] Broder J (2011). Imaging of nontraumatic abdominal conditions. Diagnostic imaging for the emergency physician.

[30] Mitsiopoulos N, Baumgartner RN, Heymsfield SB, Lyons W, Gallagher D, Ross R (1998). Cadaver validation of skeletal muscle measurement by magnetic resonance imaging and computerized tomography. J Appl Physiol.

[31] Ekström S, Malmberg F, Ahlström H, Kullberg J, Strand R (2020). Fast graph-cut based optimization for practical dense deformable registration of volume images. Comput Med Imaging Graph.

