# Supplementary material for: Voxel-wise body composition analysis using image registration of a three-slice CT imaging protocol: methodology and proof-of-concept studies
Source: Biomed Eng Online. 2024 Apr 13;23:42. doi: 10.1186/s12938-024-01235-x (PMC11015680; doi:10.1186/s12938-024-01235-x)
Supplement: Supplementary file 1 — Additional file 1: Figure S1 Visual representations of liver, abdomen, and thigh slices of templates (reference images), targeted images (moving or deforming images), and registered images (transformed images). Figure S2. Resulting Imiomics collage for 3-slice CT images of (liver, abdomen, and thigh). From left to right, selected processed CT template images, deformed Hounsfield unit, and Jacobian determinant images are represented for all male (n=502) and female (n = 455) subjects, from the SCAPIS cohort. The collage Imiomics (deformed HU and Jac determinant) images were associated with non-imaging data. The Hounsfield unit (HU) and Jacobian (Jac) collage show voxel-wise regression results (beta values) between a 3-slice image (liver, abdomen, and thigh) and the corresponding liver fat measurements in HU units. The subsequent HU and Jac images display correlations between liver CT slice and spleen area in cm2, abdomen CT slice and VAT area in cm2, thigh CT slice and thigh IMAT area in cm2. Similarly, in the last set of HU and Jac images, correlations between liver CT slice and abdominal SAT area in cm², abdomen CT slice and abdominal SAT area in cm2, thigh CT slice and thigh SAT area in cm2 are represented. Small colored boxes of green, red and white in each image indicating non imaging correlation measurements. abd is short for the abdomen. Figure S3 Resulting Imiomics collage for 3-slice CT images of (liver, abdomen, and thigh). From left to right, selected processed CT template images, deformed Hounsfield unit’s images, and Jacobian determinant images are represented for all male (n=812) and female (n=1011) subjects, from the IGT cohort. The collage Imiomics (deformed HU and Jac determinant) images were associated with non-imaging data. The 3-slice (liver, abdomen, and thigh) collage shows voxel-wise regression results (beta values) between liver area for liver slice, skeletal muscle area for abdominal slice, and thigh muscle area for thigh slice. Figure S4. Res [file 12938_2024_1235_MOESM1_ESM.docx]

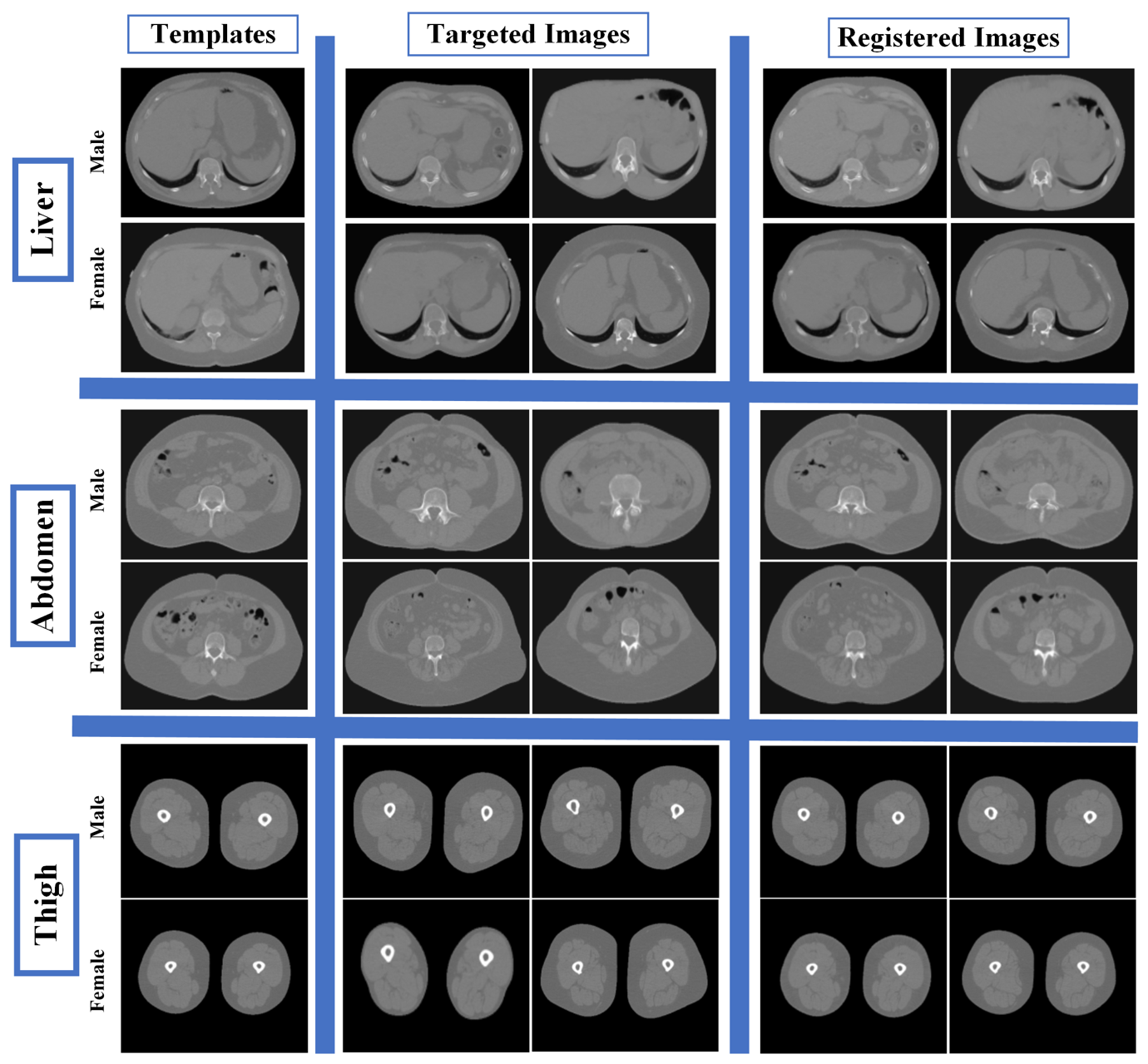


**Fig. S1** Visual representations of liver, abdomen, and thigh slices of templates (reference images), targeted images (moving or deforming images), and registered images (transformed images).


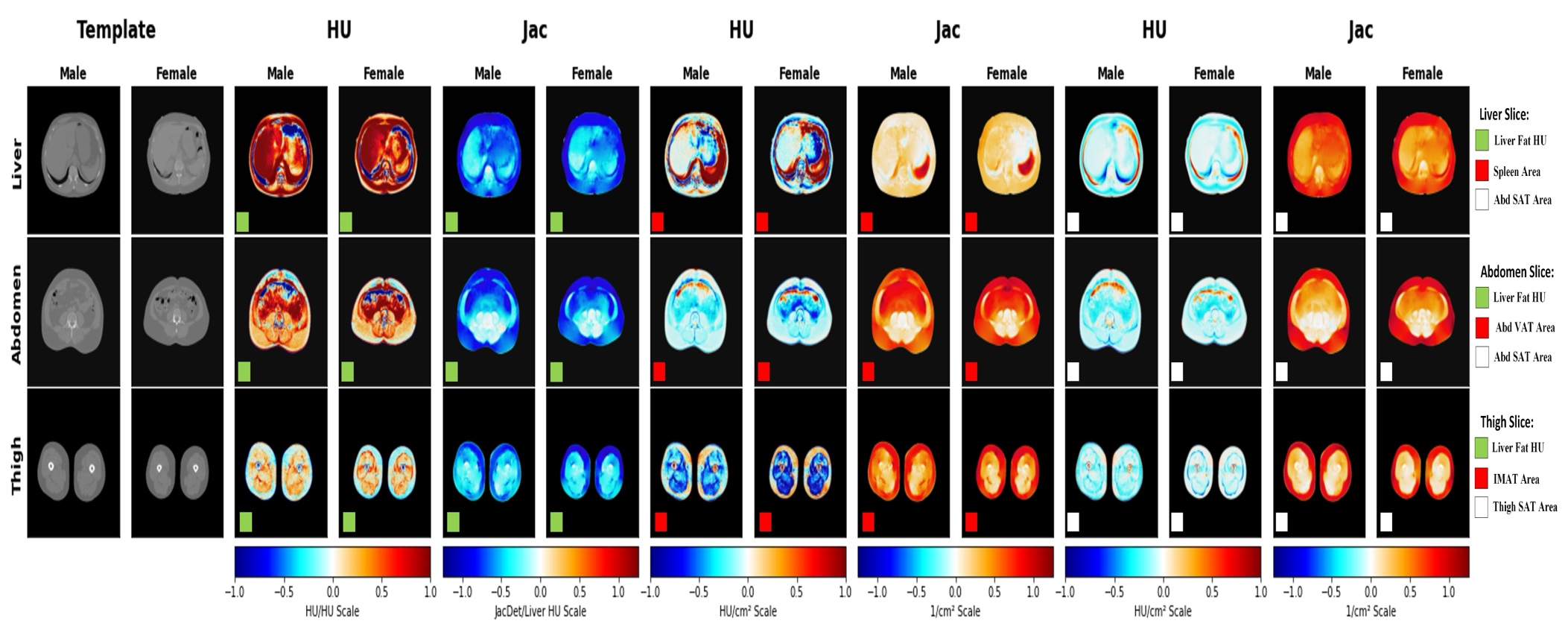


**Fig. S2** Resulting Imiomics collage for 3-slice CT images of (liver, abdomen, and thigh). From left to right, selected processed CT template images, deformed Hounsfield unit, and Jacobian determinant images are represented for all male (n=502) and female (n=455) subjects, from the SCAPIS cohort. The collage Imiomics (deformed HU and Jac determinant) images were associated with non-imaging data. The Hounsfield unit (HU) and Jacobian (Jac) collage show voxel-wise regression results (beta values) between a 3-slice image (liver, abdomen, and thigh) and the corresponding liver fat measurements in HU units. The subsequent HU and Jac images display correlations between liver CT slice and spleen area in cm², abdomen CT slice and VAT area in cm², thigh CT slice and thigh IMAT area in cm². Similarly, in the last set of HU and Jac images, correlations between liver CT slice and abdominal SAT area in cm², abdomen CT slice and abdominal SAT area in cm², thigh CT slice and thigh SAT area in cm² are represented. Small colored boxes of green, red and white in each image indicating non imaging correlation measurements. **abd** is short for the abdomen.

.


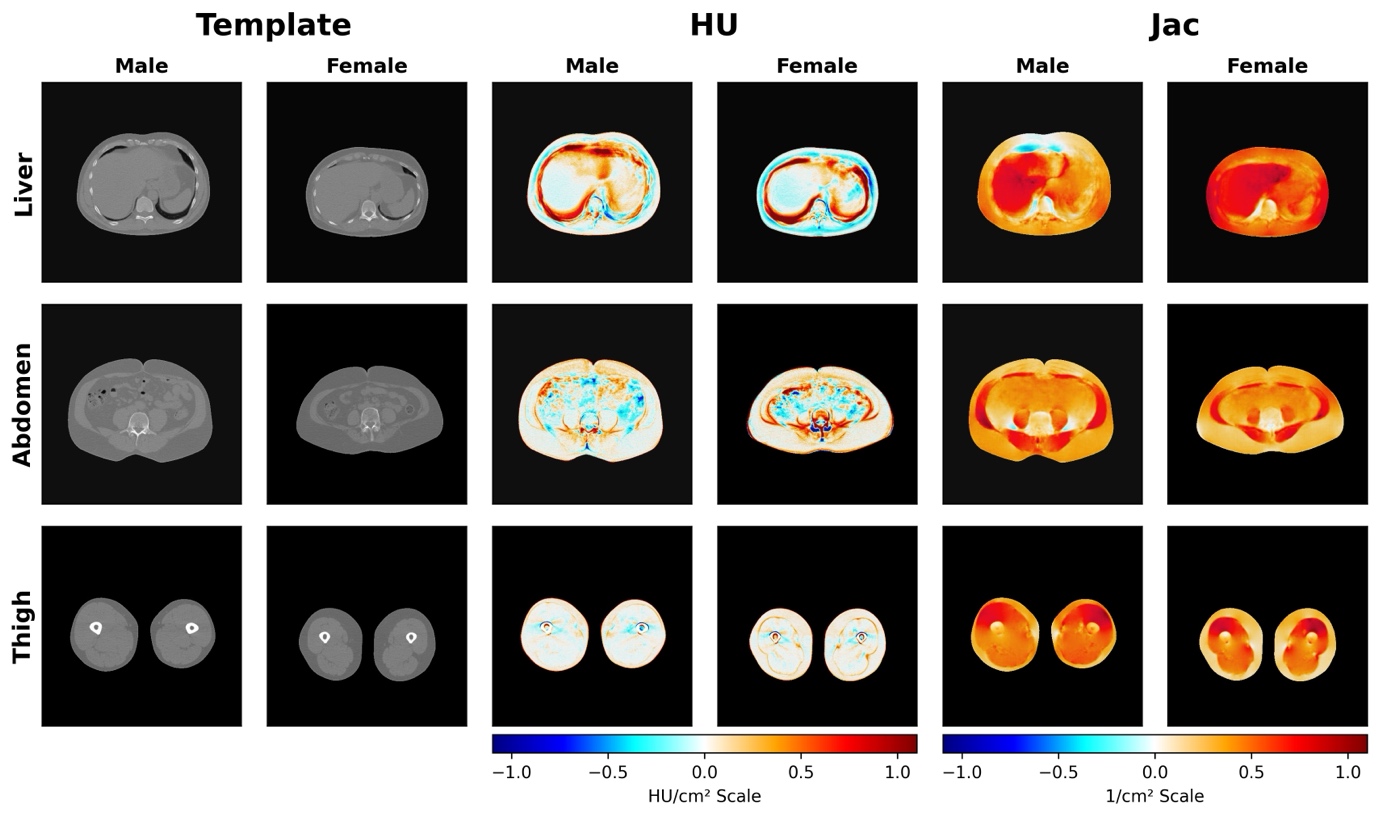


**Fig. S3** Resulting Imiomics collage for 3-slice CT images of (liver, abdomen, and thigh). From left to right, selected processed CT template images, deformed Hounsfield unit’s images, and Jacobian determinant images are represented for all male (n=812) and female (n=1011) subjects, from the IGT cohort. The collage Imiomics (deformed HU and Jac determinant) images were associated with non-imaging data. The 3-slice (liver, abdomen, and thigh) collage shows voxel-wise regression results (beta values) between liver area for liver slice, skeletal muscle area for abdominal slice, and thigh muscle area for thigh slice.


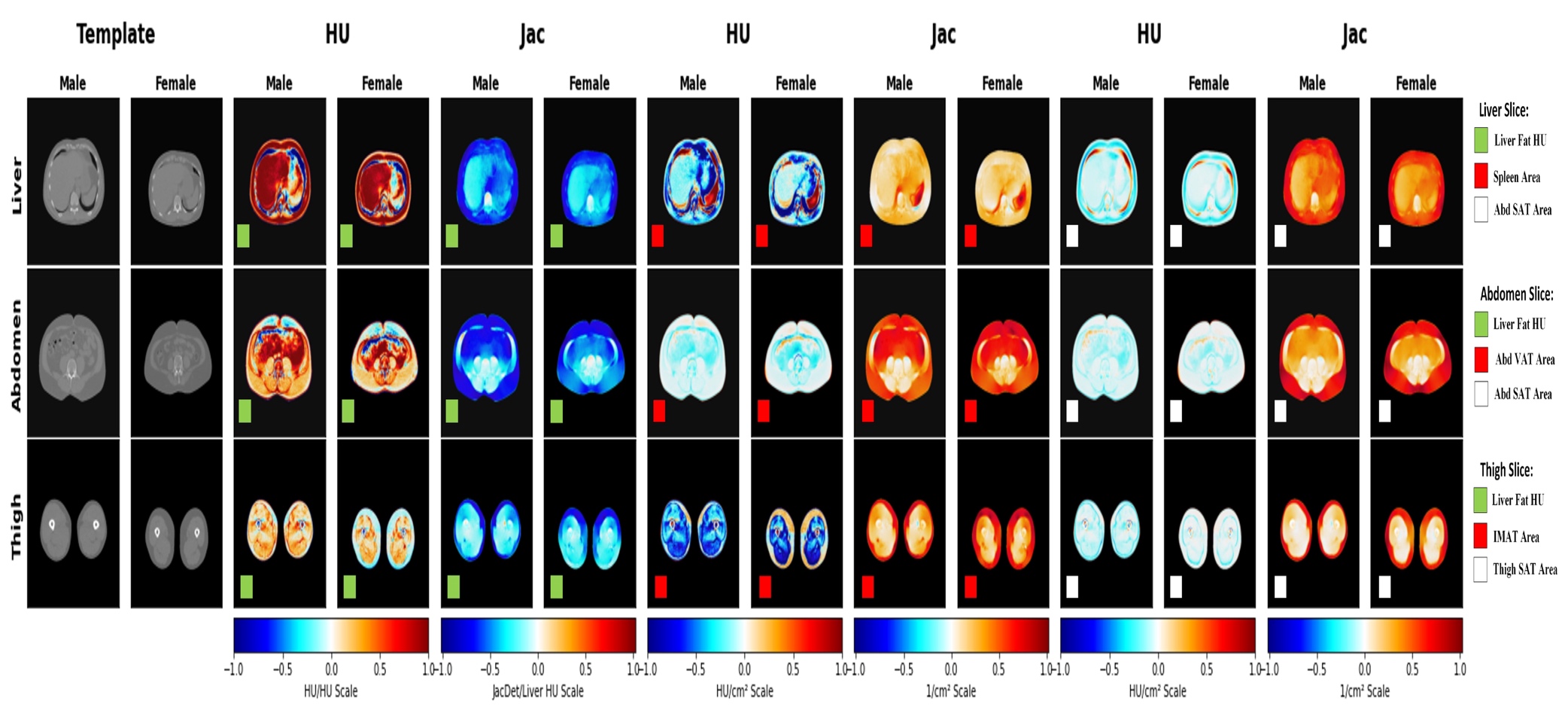


**Fig. S4** Resulting Imiomics collage for 3-slice CT images of (liver, abdomen, and thigh). From left to right, selected processed CT template images, deformed Hounsfield unit, and Jacobian determinant images are represented for all male (n=812) and female (n=1011) subjects, from the IGT cohort. The collage Imiomics (deformed HU and Jac determinant) images were associated with non-imaging data. The Hounsfield unit (HU) and Jacobian (Jac) collage show voxel-wise regression results (beta values) between a 3-slice image (liver, abdomen, and thigh) and the corresponding liver fat measurements in HU units. The subsequent HU and Jac images display correlations between liver CT slice and spleen area in cm², abdomen CT slice and VAT area in cm², thigh CT slice and thigh IMAT area in cm². Similarly, in the last set of HU and Jac images, correlations between liver CT slice and abdominal SAT area in cm², abdomen CT slice and abdominal SAT area in cm², thigh CT slice and thigh SAT area in cm² are represented. Small colored boxes of green, red and white in each image indicating non imaging correlation measurements. abd is short for the abdomen.

.


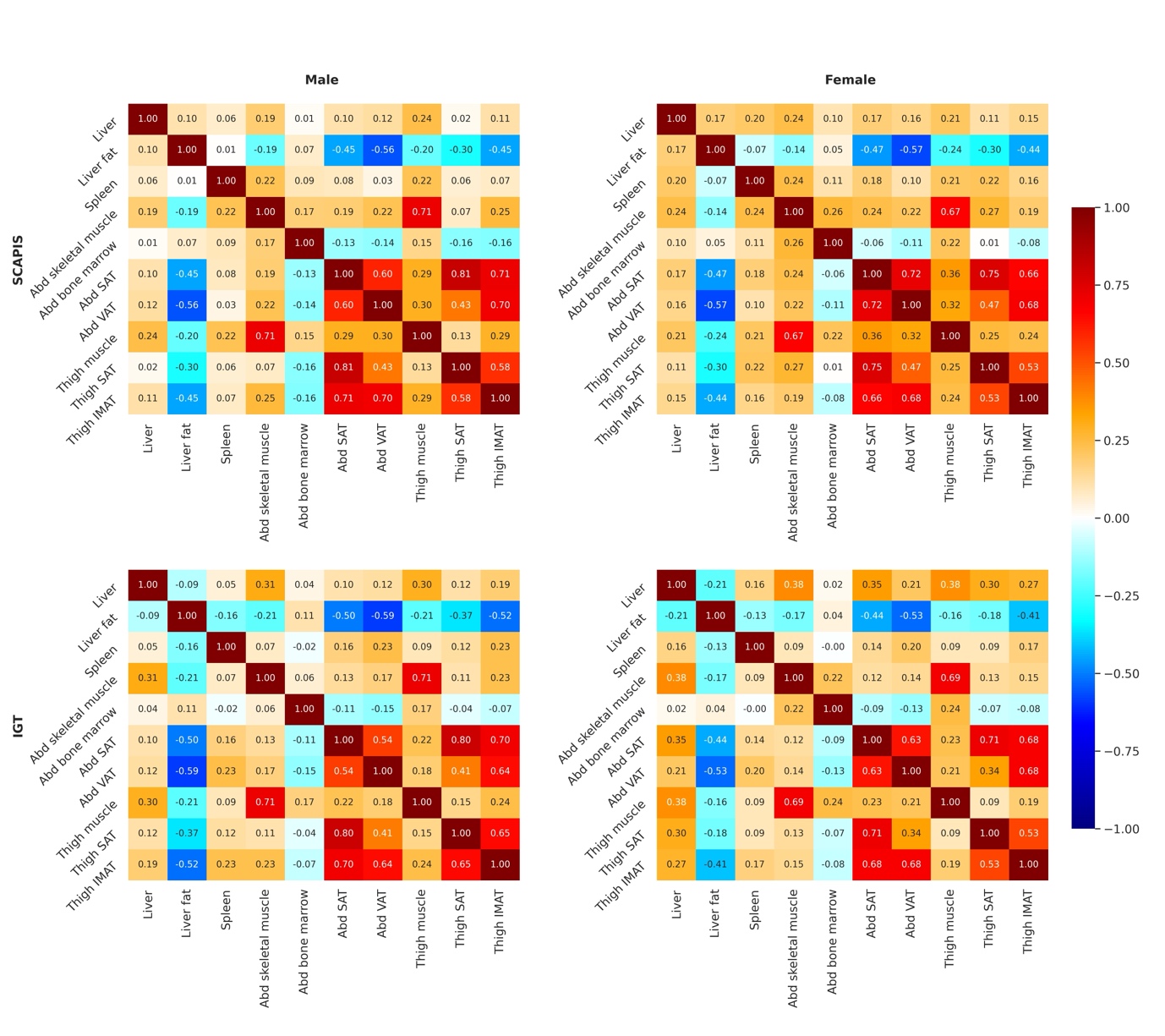


**Fig. S5** Correlation (Pearson correlation coefficient) matrix of non-imaging variables (explicit measurements in cm^2^ and HU attenuation) for male and female participants (n=8285) in SCAPIS and IGT studies. Measurements are in terms of area(cm^2^), and liver fat in terms of Hounsfield unit. **abd** is short for the abdomen.
